# Post-traumatic stress disorder and psychological distress in Chinese youths following the COVID-19 emergency

**DOI:** 10.1177/1359105320937057

**Published:** 2020-07-06

**Authors:** Leilei Liang, Tingting Gao, Hui Ren, Ruilin Cao, Zeying Qin, Yueyang Hu, Chuanen Li, Songli Mei

**Affiliations:** Jilin University, China

**Keywords:** COVID-19, negative coping style, psychological distress, PTSD, youths

## Abstract

This study aims to explore the relationship between psychological distress and post-traumatic stress disorder among Chinese participants as the result of COVID-19 outbreak. This study was conducted within 1 month after COVID-19 appeared in China, it included 570 participants age from 14 to 35. The results indicated that 12.8% of all participants with the symptoms of post-traumatic stress disorder and the effects of psychological distress on post-traumatic stress disorder was mediated by negative coping style. Gender moderated the direct effect between psychological distress and post-traumatic stress disorder, which is a significant discovery for relevant departments to take further measures.

## Introduction

At the end of 2019, a series of cases of an unfamiliar type of pneumonia has been reported in Wuhan City, Hubei Province, China. It has been confirmed that this pneumonia is caused by a new type of coronavirus, which World Health Organization has officially named it 2019 novel coronavirus disease (Du et al., 2020). On 30 January 2020, the World Health Organization announced a public health emergency of international concern (PHEIC) (WHO, 2020). This public health emergency not only damages people’s physical health, but also have a significant impact on their mental health (Huang et al., 2020; Wang et al., 2020c). Psychological distress as a broader manifestation of mental health-related problems, which is characterized by symptoms of depression anxiety, stress-related concerns and it is known to continue to show severity (Drapeau et al., 2012). In previous studies, psychological distress mainly included related psychological problems such as depression, anxiety, and stress (Higuchi et al., 2016; O’Brien et al., 2016). In a survey with regard to the mental health of general population 2 weeks after the COVID-19 outbreak in China, which the result showed about one-third of participants reported moderate to severe level of anxiety (Wang et al., 2020a), and nearly 40.4% of the youth had a tendency to have psychological problems (Liang et al., 2020). Simultaneously, medical staff also showed signs of irritability, unwillingness to rest, and difficulties in emotion management and existential stress (Chen et al., 2020; Zaka et al., 2020). In addition, the impact of infectious diseases and microbial threats on mental health has become an important public health issue (Holloway et al 1997; Norwood et al., 2001). However, most of previous studies focused on the impacts of public health emergencies such as infectious diseases on the medical staffs who are diagnosed with post-traumatic stress disorder (PTSD) (Brooks et al., 2018; Kang et al., 2020), and less on the impacts of PTSD diagnoses among younger people with lower adaptive capacities and less mature cognitive abilities which make them vulnerable against psychological distresses (Cénat and Derivois, 2014). Thus, about within 1 month after COVID-19 occurred in China, we conducted this cross-sectional study to assess the mental state of young people who are diagnosed with PTSD due to this outbreak.

PSTD is a psychological disorder which can occur after people went through a traumatic experience such as earthquakes, hurricanes and SARS (Ding and Xia, 2014; Gonzalez et al., 2019; Mak et al., 2010). Its basic feature is the characteristic symptoms result from the exposure to a traumatic experience, or a personal tragic life event, or witness events involving death, injury or threat to the physical integrity of others (Lin et al., 2007). And people with PTSD also will be forced to relive the negative effect caused by traumatic event that gives them the disorder, which can cause dramatic changes in their cognition and mood, make them avoid trauma-related stimuli at all cost, these symptoms has an important impact on daily life and work of people (Farooqui et al., 2017; Sekiguchi et al., 2013). With the extremely high infection concerns, enough evidences has demonstrated COVID-19 was considered as a life-threatening public health emergency and a disease serious enough to cause PTSD. According to the cognitive model of PTSD, the negative emotions experienced by traumatized patients (such as fear, sadness, and anger) can cause them to adopt negative assessment as a way to deal with traumatic events, which may lead to PTSD. Furthermore, previous studies have shown that participants with higher level of psychological distress, such as anxiety, depression, and fear, are more likely to develop PTSD symptoms (Wang et al., 2020b; Xi et al., 2020). This may be because people fear injury and death, especially under unexpected and unprepared situations, which can create panic, fear, and tension (Xu et al., 2016). Since the events are unexpected, people will feel confused about their current situation, and they will be uncertain about their future (Yates and Stone, 1992). Furthermore, this uncertainty may cause psychological distress in people, which would result in huge psychological stress aggravating PTSD symptoms. Therefore, psychological distress is a predictor of PTSD. However, the mediating and moderating mechanisms underlying psychological distress and PTSD need further investigation.

Studies have shown that in the absence of other adaptive coping strategies, youths may use material coping as a negative coping style (NCS) to cope with psychological distress (Acierno et al., 1996; Pollice et al., 2011; Vlahov et al., 2002). In addition, numerous empirical studies also proved that adolescents who use NCS after experienced traumatic events such as earthquakes and hurricanes have a negative impact on their PTSD symptoms (Carr et al., 1995; Pina et al., 2008;), which may be a way to alleviate the symptoms of PTSD in youths with psychological distress. It was mainly reflected in the use of methods including denial, blaming, social withdrawal, and disengagement aim to avoid the problematic situations during and after emergencies (Zheng et al., 2012). Thus, this study proposed the hypothesis that NCS would mediate the association between psychological distress and PTSD.

**Figure 1.**
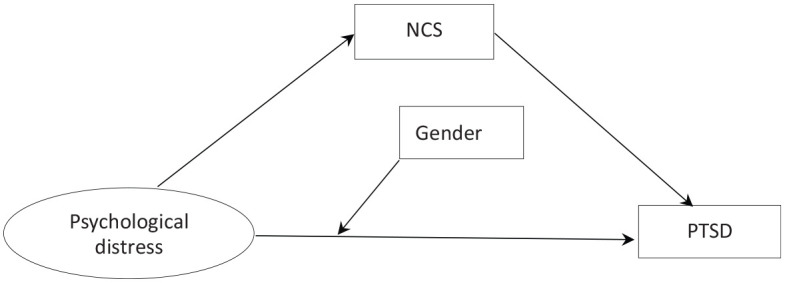
The conceptional framework of the moderated mediation model.

In addition, gender is an important biological determinant of vulnerability to psychosocial stress (Wang et al., 2007). There was no consistent conclusion regarding the relationship between gender and psychological distress and PTSD. Research indicates that compare to males, females show more PTSD symptoms (Kun et al., 2013; Qi et al., 2020). When an emergency occurs, women may be more vulnerable than men, less likely to use effective coping strategies, and tend to interpret PTSD negatively (Tolin and Foa, 2006). On the other hand, women are instinctively more sensitive to loss and stress, and therefore may develop negative emotions and PTSD symptoms (Dell’Osso et al., 2011). However, some studies have found that men have more PTSD diagnoses than women (Du et al., 2018; Liang et al., 2020), this may be because the men take more responsibility in taking care of the family (Guo et al., 2017), therefore show more symptoms of PTSD. These different results inspired us to further explore the relationship between gender, psychological distress and PTSD. Thus, this study proposes the hypothesis that the gender would moderate the direct association between the psychological distress and PTSD (Figure 1).

## Method

### Participants and procedures

A cross-sectional study was designed in the first month since COVID-19 outbreak hits China. The study took a snowball sampling approach to collect questionnaires remotely, targeting people age from 14 to 35. The questionnaires will be sent to participants through a well-known smartphone social network application called WeChat. Participants can share their questionnaires with their friends, who can then share with their friends, as a way to expand the sample size. Finally, approximately 600 questionnaires were collected from participants. After deleting the duplicate IDs and random questionnaires, this study collected a total of 570 valid questionnaires, the valid response rate was 95.0%. Before collecting the data, the participant was given an informed consent, and researchers received a verbal consent from the participant in return.

### Measures

#### The PTSD Checklist-Civilian Version

The diagnosis of PTSD was done by using the PTSD Checklist-Civilian Version (PCL-C) (Weathers et al., 1993), which was designed to assess participants’ responses to traumatic experiences encountered in daily lives. The severity of PTSD symptoms was measured using a five-point Likert scale. The total score range from 17 to 85, with higher score indicates more serious symptomatological PTSD and participants with a cut-off score of 38 or higher were diagnosed with PTSD symptoms (Grubaugh et al., 2007). This scale was widely used to evaluate symptomatic PTSD of Chinese adolescents, with high reliability and validity (Yang et al., 2007). An early study using this threshold reported Cronbach’s alpha of the whole scale was 0.94 (Zhou et al., 2015). In this study, the Cronbach’s alpha of PCL-C was 0.943.

#### The General Health Questionnaire Scale

The psychological distress was measured using the General Health Questionnaire (GHQ-12) (Goldberg et al., 1997), which contains 12 items assessing participant’s mental health. It uses a four-point Likert scale, which higher score indicates higher degree of disturbance in the mind. The questionnaire has been proven with great reliability and validity, and it is commonly used by different Chinese research (Fares et al., 2016). Previous research has shown that the GHQ-12 coefficients ranged from 0.78 to 0.95 (Arnberg et al., 2012; Yusoff et al., 2010). In the present study, the Cronbach’s alpha of GHQ-12 was 0.79.

#### Simplified Copying Style Questionnaire

The Simplified Coping Style Questionnaire (SCSQ) (Xie, 1998) was a 20-item self-report scale, including two sub-scales: positive coping (12 items) and negative coping (eight items). Each item options ranged from 0 (never) to 3 (very frequently), and the scores on corresponding sub-scale indicates the level of coping strategy everyone possesses, this study selects the subscale of negative coping style in this questionnaire. Previous study has showed that the Cronbach’s alpha of negative coping was 0.78 (Lin et al., 2020). In the present study, the Cronbach’s alpha of negative coping was 0.83.

### Data analysis

In this study, we conducted a descriptive analysis to describe the basic sociodemographic characteristics of participants, and a correlation analysis to verify the relationship between variables. We also used multiple linear regression analysis via SPSS 24.0 (IBM Corp) and a PROCESS macro to tests the mediating effect of NCS and the moderating effect of gender between psychological distress and PTSD. Finally, this study conducted 95% bootstrap confidence intervals (CI) based on 5000 bootstrapped samples, with the effects being significant when the results did not include zero. A significance level of *p <* 0.05 was used for all variables.

## Results

### Participant characteristics

As the result, the demographic characteristics of 570 participants are shown in Table 1. A total of 205 males (36.0%) and 365 females (64.0%) were included. The age range of all participants was between 14 and 35 years old. The majority of participants aged 21 to 30 years old (73.3%). As for the education level, a significant proportion of participants were undergraduates (78.2%), follow by master’s degree or above (13.9%). About 69.1% of all participants have a monthly income less than 2000 RMB. See Table 1 for more information.

**Table 1.** Demographic characteristics of the participants (*n* = 570).

Variables	*n*	%
Gender		
Male	205	36.0
Female	365	64.0
Age		
⩽20	131	23.0
21–30	418	73.3
⩾31	21	3.7
Educational level		
High school or secondary school	45	7.9
Undergraduate or college	446	78.2
Master’s degree or above	79	13.9
Monthly income		
⩽2000 RMB	394	69.1
2001–3000 RMB	79	13.9
3001–4000 RMB	44	7.7
4001–5000 RMB	15	2.6
⩾5001 RMB	38	6.7

### Preliminary analyses

Table 2 presented the means, standard deviations and bivariate correlations of all study variables. The results indicated that both psychological distress and NCS had significant positive correlation with PTSD.

**Table 2.** Descriptive statistics and correlation among variables.

Variables	1	2	3	4
Gender	1			
The psychological distress	−0.07	1		
PTSD	−0.09*	0.37**	1	
NCS	−0.09*	0.16**	0.28**	1
M	1.64	11.73	27.32	19.53
SD	0.48	6.25	10.42	5.38

Gender was coded so that 1 = male and 2 = female.

* *p <* 0.05; **p < 0.01.

### Testing for mediation effect

This study used multiple liner regression analysis (Baron and Kenny, 1986) to test whether NCS mediate the association between the psychological distress and PTSD. In this study, the direct path coefficient from the psychological distress to PTSD was significant (*B* = 0.61, *β* = 0.37, *p* < 0.001), and the psychological distress was also significantly associated with NCS (*B* = 0.13, *β* = 0.15, *p* < 0.001). When we considered both the psychological distress and NCS as predictors of PTSD in the regression model, the path coefficients of the psychological distress on PTSD remained significant (*B* = 0.55, *β* = 0.33, *p* < 0.001). In addition, we used the PROCESS macro (Model 4) in SPSS and perform the bootstrap method to test the indirect effect (Preacher and Hayes, 2008). The results indicated that the psychological distress on PTSD through NCS was significant (95% CI = [0.02, 0.10]; excluding 0). Thus, this study indicated that NCS mediated the association between psychological distress and PTSD.

### Testing for the moderated mediation effect

**Figure 2.**
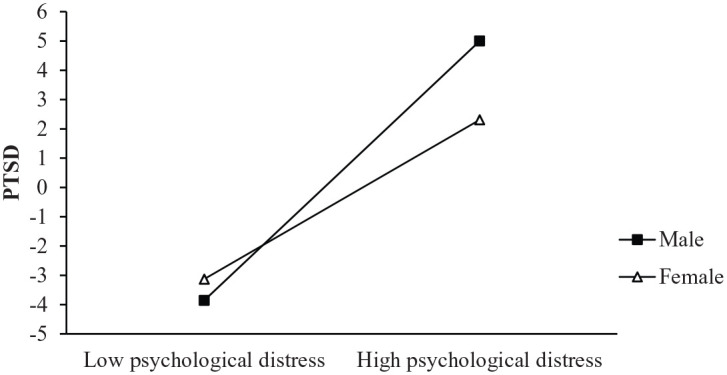
Gender moderates the effect of the psychological distress on PTSD.

Before examining the moderated mediation analysis, all the variables were mean centered to minimize multicollinearity. Table 3 showed the detailed results. In Model 1, the psychological distress was positively related to PTSD (*β* = 0.35, *p* < 0.001). Gender did not relate to PTSD (*β* = −0.06, *p* > 0.05), but the interaction term between the psychological distress and gender was positively related to PTSD (*β* = −0.08, *p* < 0.05), which indicated that gender could moderate the association between psychological distress and PTSD. In Model 2, the main effect of psychological distress on NCS was significant (*β* = 0.15, *p* < 0.001), but this effect could not be moderated by the gender (*β* = −0.01, *p* > 0.05). In model 3, the main effect of NCS on PTSD was significant (*β* = 0.22, *p* < 0.001), however, this effect could not be moderated by the gender (*β* = −0.02, *p* > 0.05). Simple slope analyses were used to further analyze the moderate effect of gender on the relationship between psychological distress and PTSD (see Figure 2). The results indicated that psychological distress can be significantly associated with PTSD in males (*β*_simple_ = 0.71, *t* = 7.17, *p* < 0.001) and females (*β*_simple_ = 0.44, *t* = 5.25, *p* < 0.001). The effect of psychological distress on PTSD was higher in males than females.

**Table 3.** Testing the moderated mediation effect of psychological distress on PTSD.

Independent variables	Model 1 (PTSD)		Model 2 (NCS)		Model 3 (PTSD)	
	*β*	*t*	*β*	*t*	*β*	*t*
The psychological distress	0.35	9.01***	0.15	3.56***	0.33	8.56***
Gender	−0.06	−1.06	−0.08	−1.86	−0.05	−1.23
The psychological distress × Gender	−0.08	−2.01*	−0.01	−0.09		
NCS					0.22	5.48***
NCS × Gender					−0.02	−0.71
R^2^	0.14		0.03		0.18	
F	32.09***		5.86**		32.42***	

* *p* < 0.05; **p < 0.01; ***p < 0.001.

In addition, this study conducted the PROCESS macro method (Model 5) to further analyze the moderation mediation, which was able to verify the above assumptions. The index of moderated mediation was –0.27 (SE = 0.13, 95%CI = [–0.526, –0.021]). Analysis of the moderation effect indicated that this path was significantly for males (95% CI = [0.515, 0.904]; excluding 0) and females (95% CI = [0.272, 0.599]; excluding 0), which tested the above assumption.

## Discussion

In this study, we found that within 1 month after the occurrence of COVID-19 outbreak in China, the prevalence of PTSD was 12.8%, which was lower than a cross-sectional study of the prevalence of PTSD (22.3%) 1 month after the earthquake in Chengdu, Sichuan province, China (Lau et al., 2010) and also lower than PTSD (41%) reported by SARS patients 3 months after discharge (Kwek et al., 2006). However, in a 4-year follow-up study of discharged SARS patients, the result shows the reported cases of PTSD was 44.1% among all patients (Hong et al., 2009). And in a report from Italy, the prevalence of PTSD 8 years after the earthquake is only 0.5% (Priebe et al., 2011). The above differences in PTSD prevalence after a traumatic event may be due to the differences in research methods, culture, type and severity of the disaster, time interval measured after the disaster and diagnostic criteria (Liu et al., 2017). In addition, a systematic review demonstrated that the PTSD rate declined after disaster (Dai et al., 2016), but studies also pointed out that adolescents affected by traumatic events were prone to invasive thoughts such as sleep disorders, nightmares, and separation anxiety (Yule, 2001). Thus, this emphasizes that relevant government agencies should take measures aimed at the mental health of youths as soon as a public health emergency occurs. In addition, related preventive and clinical measures should also be applied to prevent and treat the damage of COVID-19 to participants’ health. A recent study suggested that COVID-19 can cause nervous system damage (Wu et al., 2020), and also indicated that the negative mental states (such as depression and anxiety) are relate to changes in the immune system (Rajkumar, 2020). Thus, from the perspective of psychoneuroimmunology, the immune system can be improved by eliminating daily psychological distress, maintaining good sleep quality, balancing nutrition intake, keeping a healthy lifestyle and exercising regularly. As the result, if people have strong immune systems, it can reduce the risk of COVID-19 infection (Kim and Su, 2020; Matias et al., 2020; Ng et al., 2018). Also, this study provides references that are significant for relevant clinical researches, and they can help psychiatrists to effectively identify the groups with mental health issues due to the COVID-19 outbreak (Zhou et al., 2013). After picking out people in need, psychological professionals can provide remote services like telephone and internet, which can speed up the development of technologies along the way, such as electronic consent forms and telemedicine (Smith et al., 2020).

As expected, the effect of psychological distress on PTSD was mediated by NCS, which supported the initial hypothesis. In the study, the mediation analysis indicates that psychologically distressed adolescents are more likely to engage in negative coping strategies, which ultimately lead to PTSD (Vlahov et al., 2002). In addition, traumatic experiences cause people to experience more negative emotions, which in turn results in PTSD (Quan et al., 2020). People overwhelmed with negative emotions tend to choose negative behaviors like self-blame or avoid problems (Xiang et al., 2016), this may be because negative emotions have been theorized as an obstacle mechanism to affect NCS (Folkman and Lazarus, 1988). Such actions can further weaken people’s capability to deal with psychological distresses, which will eventually lead to mental illnesses like PTSD (D’Amico et al., 2013). Moreover, studies have also shown that the symptoms of PTSD and psychological distress have overlapping characteristics (Hurlocker et al., 2018), sharing common characteristics such as inattention, hypervigilance, and emotional disorders (Pacella et al., 2013), in which may be because the related symptoms didn’t occur in isolation (Borsboom and Cramer, 2013). Therefore, these results may help psychiatrists and psychologists to develop or take interventions that target specific symptoms of this relationship. When a public health emergency occurs, the local government should immediately provide relevant psychological interventions to help young people overcome negative emotional experiences, because early psychological interventions can help reduce the prevalence of PTSD (Zhou et al., 2013). On the other hand, during the COVID-19 outbreak, the government and other relevant agencies should encourage adolescents to take active coping styles, enhance their ability of learning from difficult situations and actively seek help from others who can protect them from PTSD (Liu et al., 2016).

Our study indicated that gender played a moderating role in the direct effect between the psychological distress and PTSD, which supported the hypothesis. With the increase of psychological distress, the prevalence of women PTSD increased significantly, but the prevalence of men PTSD increase more. From a biological perspective, gender is an important biological determinant of the vulnerability of psychological distress, and gender differences have been identified in the brains’ activation of stress. This may be because by examine the brain activity in response to physiological stresses, significant differences appear between men and women. When people try to cope with psychological distress, the activities of prefrontal lobe in males’ brains are asymmetric, meanwhile, the activities are mainly focus on limbic system for females. In brief, the results show men and women will choose different actions and coping strategies in response to when people try to cope with psychological distress (Wang et al., 2007). The reason may also be caused by the different coping styles during the study time, or it may be because women are more likely to show symptoms during emergencies, which can effectively reduce the chance of get PTSD (Du et al., 2018). In addition, social expectations related to gender roles may lead to differences (Tolin and Foa, 2006). In China, there may be such a basic rule that men are normally perceived as powerful figures who are dominant in status and rights (Chen et al., 2009), but this social trend may also bring more psychological distresses to men. During the occurrence of COVID-19, the Chinese government has implemented the strictest prevention and control measures, people need isolation at home to prevent infection, on the other hand, adult males will experience more psychological distress due to increasing financial pressures and loss of job opportunities. Such high stress situations can increase chances of getting PTSD in males. Therefore, this study suggests that corresponding measures should be taken based on the gender differences in the PTSD.

### Limitations

Certain limitations of this study should be recognized. We examined only general psychopathology using the GHQ-12 rather than a specific mental health problem (such as depression and fear). Our study used a cross-sectional design, which cannot provide strong evidence for causality. Thus, further research should use a longitudinal design. In addition, our study is limited by sample size. In order to get more detailed results, larger and more universal sample groups are needed.

## Conclusion

This study was conducted within 1 month of the COVID-19 emergency in China. In this study, 12.8% of participants were diagnosed with PTSD, which indicates the significance of the public health emergency. Government and other relevant agencies must take swift and systematic action to improve the mental health of youth. This study found that general mental health can be affected by PTSD through NCS, highlighting the moderating effect of gender on this association. The prevalence of PTSD in women increased significantly with psychological distress, but the prevalence of men PTSD increased even more. This study provides a reference for formulating psychological intervention measures to improve people’s mental health and psychological adaptability during the occurrence of COVID-19 and any similar pandemics in the future.
